# Machine Perfusion for Human Heart Preservation: A Systematic Review

**DOI:** 10.3389/ti.2022.10258

**Published:** 2022-03-21

**Authors:** Guangqi Qin, Victoria Jernryd, Trygve Sjöberg, Stig Steen, Johan Nilsson

**Affiliations:** ^1^ Department of Clinical Sciences Lund, Cardiothoracic Surgery, Lund University and Skane University Hospital, Lund, Sweden; ^2^ Department of Translational Medicine, Thoracic Surgery and Bioinformatics, Lund University, Lund, Sweden

**Keywords:** review, heart transplantation, machine perfusion, heart preservation, donor

## Abstract

Currently, static cold storage (SCS) of hearts from donations after brainstem death remains the standard clinically. However, machine perfusion (MP) is considered an approach for donor organ management to extend the donor pool and/or increase the utilization rate. This review summarizes and critically assesses the available clinical data on MP in heart transplantation. We searched Medline (PubMed), Cochrane, Embase, and clinicaltrials.gov, along with reference lists of the included publications and identified 40 publications, including 18 articles, 17 conference abstracts, and five ongoing clinical trials. Two types of MP were used: hypothermic MP (HMP) and normothermic MP (NMP). Three studies evaluated HMP, and 32 evaluated NMP. Independent of the system, MP resulted in clinical outcomes comparable to traditional SCS. However, NMP seemed especially beneficial for high-risk cases and donation after circulatory death (DCD) hearts. Based on currently available data, MP is non-inferior to standard SCS. Additionally, single-centre studies suggest that NMP could preserve the hearts from donors outside standard acceptability criteria and DCD hearts with comparable results to SCS. Finally, HMP is theoretically safer and simpler to use than NMP. If a machine malfunction or user error occurs, NMP, which perfuses a beating heart, would have a narrower margin of safety. However, further well-designed studies need to be conducted to draw clear conclusions.

## Introduction

Heart transplantation is the most effective method used to treat end-stage heart disease. Currently, static cold storage (SCS) of hearts from donations after brainstem death (DBD) remains the standard practice. SCS combines cardioplegia and hypothermia, which can significantly reduce the energy demand of the donor heart (1). However, despite decades of effort, the cold ischemia time has been limited to 4–6 h. Prolonged cold ischemia and ischemia-reperfusion injury (IRI) have been recognized as significant causes of post-transplant graft failure. According to the International Society for Heart and Lung Transplantation registration, the survival rate decreases as the ischemic time increases (2). The continuous shortage of donor hearts has always been a major limiting factor for heart transplantation (3).

Machine perfusion (MP) is considered an ideal approach for donor organ management to extend the donor pool and/or increase the utilization rate. Perfusion can supply the metabolic need of the myocardium, thus minimizing irreversible ischemic cell injury and death. Several heart perfusion systems, which are either hypothermic MP (HMP) or normothermic MP (NMP), have successfully preserved animal and/or human hearts (4). The longest reported successful human heart preservation time was 16 h with NMP (5). Currently, there is only one commercially available perfusion system for clinical use, the organ care system (OCS), and one recently tested system, the non-ischemic heart preservation system (NIHP) (6, 7). Another approach to extend the donor pool is to utilize organs from donation after circulatory death (DCD) (4, 8). For these donor hearts, MP can provide a platform to resuscitate, preserve, assess and even possibly recondition the cardiac function prior to planned transplantation.

Well-designed machine perfusion can theoretically expand the donor pool in different ways. A prolonged safe preservation time allows to utilize remote donor hearts and functional assessment allows to utilize some of the DCD and high-risk donor hearts. Pediatric heart transplantation may have an extra benefit since pediatric donor shortage is even worse, and long transport time occurs more frequently.

Despite the growing number of human donor hearts preserved with MP, it remains controversial whether MP is superior to SCS. In this systematic review, we summarize and critically assess all available clinical data on MP of adult donor hearts, highlighting its therapeutic potential as well as the current limitations and shortcomings.

## Methods

### Search Strategy and Data Sources

This systematic review was performed according to the Preferred Reporting Items for Systematic Reviews and Meta-Analysis guidelines. The literature search consisted of two parts: searching for published studies and searching for ongoing clinical trials (inception to 27 June 2020). Published studies were searched in the Medline (PubMed), Cochrane, and Embase databases. The following searching terms were used in combination with AND or OR: heart transplantation, organ perfusion, *ex vivo* perfusion, *ex vivo* reperfusion, heart perfusion, cardiac perfusion, non-ischemic heart preservation, perfusion preservation, antegrade perfusion, and machine perfusion. Ongoing clinical trials were searched in clinicaltrials.gov using the term of heart transplantation for condition or disease in combination with preservation or perfusion for other terms. Only original publications in English were considered. All questions regarding the literature search and article selection were resolved by discussion between two independent reviewers. All references listed in the selected articles were screened for any further publications that were not identified in the initial search.

### Inclusion and Exclusion Criteria

Articles reporting the outcome of MP in donor hearts during primary adult heart transplantation were included. Reports that met any of the following criteria were excluded: 1) irrelevant topics, 2) duplicated data, 3) non-English language, 4) not transplanted, 5) not human, 6) pediatric, or 7) reviews, editorials, and letters to the editor.

## Results

**FIGURE 1:**
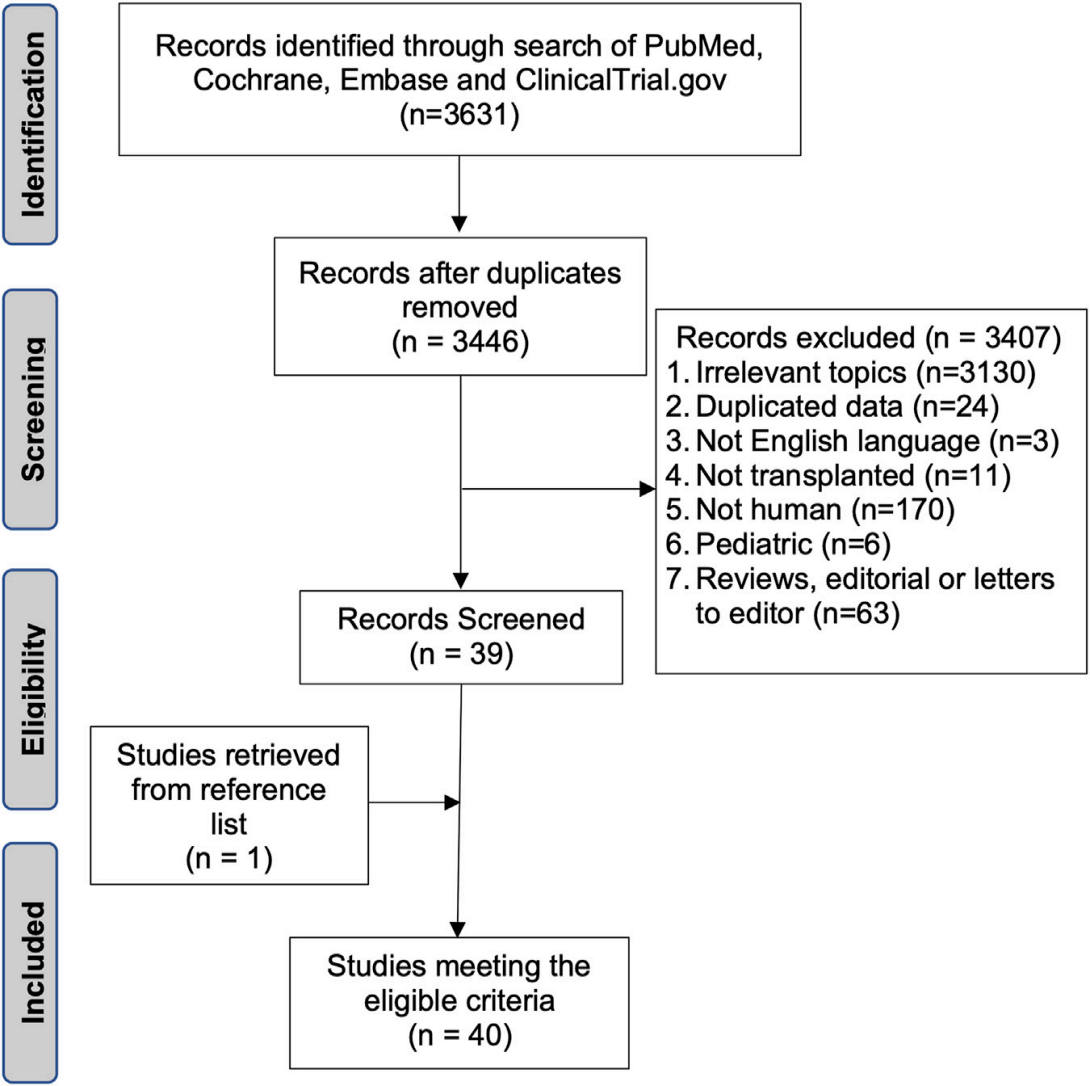
Flowchart of the search strategy.

The initial search yielded 3,446 potentially relevant records. Figure 1 shows a flowchart of the study selection process. Screening resulted in 39 relevant studies. One additional study was identified from the screening of reference lists in the included publications. Ultimately, 40 studies were included in this review: 18 papers (6, 7, 9-24), 17 conference abstracts (5, 25–40), and five ongoing clinical trials (41-45). Three studies reported multicenter data (7, 25, 40), and three were randomized controlled studies (7, 12, 13).

In clinical practice, two types of MP have been used to preserve donor hearts: HMP and NMP. The system temperature was controlled below 10°C during HMP, in contrast to 34°C during NMP. We identified three non-randomized, single-centre studies that used in-house designed HMP systems (Table 1) (6, 9, 11). Wicomb et al. demonstrated the first system for HMP of the human heart (9). In this study, four hearts were perfused with an oxygen- and carbon dioxide-bubbled crystalloid cardioplegic solution at a pressure of 8–10 cm H_2_O. All four hearts were transplanted after a total preservation time of 6, 7, 12, or 15 h. Only one patient survived after 16 months with normal heart function (9). Hill et al. reported successful heart transplantation with HMP using a colloid cardioplegic solution to perfuse eight hearts with a low flow rate (17 ml per 100 g per hour) for 221 min. For comparison, 13 hearts were preserved with cardiosol (185 min) and 50 hearts with modified St. Thomas solution (187 min). The 7-year survival rate was 70% in the St. Thomas solution group and 100% in the other two groups (11). In the third study, Nilsson et al. preserved six hearts using NIHP with a perfusion pressure of 20 mm Hg at 8°C. The perfusate comprised a hyperoncotic cardioplegic nutrition solution supplemented with hormones and erythrocytes. These six NIHP transplantations were compared with 25 SCS transplantations during the same period. The median total preservation time was longer for the NIHP group (223 min; IQR, 202–263) than for the SCS group (194 min; IQR, 164–223). The primary outcome showed a 100% event-free 6-month survival rate for NIHP recipients, compared to 72% for SCS recipients. Furthermore, creatine kinase-muscle/brain, assessed 6 h after ending perfusion, was 76 ng/ml for NIHP compared with 138 ng/ml for the SCS recipients (non-significant), indicating less myocardial damage when using the NIHP method (6).

**TABLE 1:** Hypothermic machine perfusion.

Study	Number of patients	Temperature (°C)	Perfusate	Outcome	Publication type
Wicomb et al., 1984 (9)	HMP = 4	4–10	Crystalloid cardioplegic solution	Total preservation time 12, 7, 15, and 6 h. One patient survived over 16 months	Single-center
Hill et al., 1997 (11)	HMP = 8, SCS = 12	Ice-cooling	Colloid cardioplegic solution	7-year survival rate 100% in both the HMP and the SCS groups	Single-center
Nilsson et al., 2020 (6)	HMP = 6, SCS = 25	8	Albumin-rich solution with erythrocytes	6-month event-free survival rate 100% in the HMP group and 72% in the SCS group	Single-center

HMP, hypothermic machine perfusion; SCS, static cold storage.

The only NMP system for clinical heart transplantation is currently the OCS. With the OCS, oxygenated donor blood is used to perfuse coronary arteries at a temperature of 34°C with a perfusion pressure of 60–90 mmHg. Lactate concentration is monitored to verify that adequate perfusion is achieved and if it is above 5 mmol/L, the heart is discarded (7). In the PROCEED II trial, five donor hearts were discarded, four because of rising lactate concentrations and one because of technical issues (7).

Twenty-one publications, including eight papers (7, 10, 12–16, 21) and 13 conference abstracts (5, 25–35, 40) presented results from using the OCS at transplantation of DBD hearts with or without a control group (Tables 2, 3). Three of these studies were randomized (Table 2). The only randomized and multicenter study, PROCEED II, which recruited 130 patients from 10 heart transplant centres in the United States and Europe, showed no significant differences in the primary endpoint (30-day patient and graft survival) or secondary endpoints. However, the mean total out-of-body time was significantly longer in the OCS group than in the control group (324 vs. 195 min) (7). The other two randomized studies reported data from single institutional heart transplant candidates, previously enrolled in the PROCEED II study and subsequently followed for an additional one and 2 years (12, 13). There were no significant differences between the OCS and SCS groups regarding changes in intimal thickness for the left main and left anterior descending coronary arteries (13). Chan et al. followed the recipient for 2 years and did not find any significant differences in patient survival, freedom from non-fatal major cardiac events, or cardiac allograft vasculopathy (12).

**TABLE 2:** Studies of normothermic machine perfusion for hearts from donation after brainstem death with static cold storage as the control group.

Study	Number of patients	Total preservation time (min)	Outcomes	Publication type	Risk case
Ardehali et al., 2015 (7)	OCS = 67, SCS = 63	OCS = 324, SCS = 195	No difference in 30-day survival rate and SAE between groups	Multi-center, randomized, article	No
Chan et al., 2017 (12)	OCS = 19, SCS = 19	OCS = 361, SCS = 207	2-year patient survival rate: 72.2% in OCS group, 81.6% in SCS group (*p* = 0.38)	Single-center, randomized, article	No
Sato et al., 2019 (13)	OCS = 5, SCS = 13	OCS = 362, SCS = 183	ΔMIT ≥0.5 mm with no significant difference between groups. From baseline to 1 year post-transplant, ΔMIT, maximal intimal area, and percent stenosis were similar between groups	Single-center, randomized, article	No
Botta et al., 2017 (26)	OCS = 7, SCS = 95	OCS = 296, SCS = 187	No significant difference in CK-MB post- transplant	Conference abstract	Yes
Falk et al., 2019 (27)	OCS = 16, SCS = 24	Not reported	OCS perfusion reduces IRI at the cytokine and endothelial level in recipient blood immediately after transplantation	Conference abstract	Not mentioned
Fujita et al., 2018 (28)	OCS = 29, SCS = 169	Not reported	Survival rate similar between groups	Conference abstract	Not mentioned
Garcia et al., 2015 (29)	OCS = 15, SCS = 15	OCS = 373, SCS = 204	30-day survival rate: 100% in OCS group and 73.3% in SCS group (*p* = 0.03)	Conference abstract	Yes
Jain et al., 2017 (14)	OCS = 1, SCS = 1	OCS = 495, SCS = 412	Total cost of OCS transplantation significantly less than SCS transplantation	Article	Yes
Koerner et al., 2014 (15)	OCS = 29, SCS = 130	OCS = 313, SCS: not reported	No significant difference in cumulative survival rates at 30 days, 1 year, and 2 years	Article	No
Rojas et al., 2020 (30)	OCS = 49, SCS = 48	OCS = 402, SCS = 225	No significant difference in 30-day, 1-year, and 2-year survival rate	Conference abstract	Yes
Sponga et al., 2019 (31)	OCS = 17, SCS = 70	Not reported	Improved 30-day, 1-year, and 5-year survival rate in the OCS group	Conference abstract	Yes
Sponga et al., 2020 (25)	OCS = 44, SCS = 21	OCS = 428, SCS = 223	No significant difference in 30-day mortality	Conference abstract	Yes

IRI, ischemia-reperfusion injury; MIT, maximal intimal thickness; NS, not significant; OCS, organ care system; SAE, serious adverse events; SCS, static cold storage.

**TABLE 3:** Non-randomized studies of normothermic machine perfusion for hearts from donation after brainstem death, without control group.

Study	Number of patients	Total preservation time (min)	Outcomes	Publication type	Risk case
Ayan Mukash et al., 2019 (32)	47	Not reported	Kaplan-Meier survival estimates 91%, 85%, and 80% at 3 months, 6 months, and 1 year	Conference abstract	Yes
Garcia et al., 2016 (33)	60	Not reported	Survival rate similar between regular donor group (*n* = 24) and extended criteria donor group (*n* = 36)	Conference abstract	Yes
Garcia et al., 2014 (16)	26	371	Survival rate 100% at 1 month and 96% at follow-up of 257 days	Article	Yes
Kaliyev et al., 2019 (10)	43	344	30-day survival 100%	Article	Not mentioned
Koerner et al., 2012 (34)	13	Not reported	1- and 2-year survival rate 89%	Conference abstract	Not mentioned
Nurmykhametova et al., 2018 (5)	1	960	Total out-of-body time 16 h, longest out-body time to date	Conference abstract	Yes
Rojas et al., 2020 (40)	76	382	Survival rate 92.1% and 82.9% at 30 days and 1 year	Conference abstract	Yes
Stamp et al., 2015 (21)	1	611	Total out-of-body time 10 h	Article	Yes
Yeter et al., 2014 (35)	21	388	Freedom from cardiac-related death 95% at 30 days and 6 months, 87% at 1 and 4 years	Conference abstract	Yes

Thirteen studies (5, 14, 16, 21, 25, 26, 29–33, 35, 40) used the OCS in high-risk cases. High risk was defined as an adverse donor/recipient profile, including an estimated ischemic time longer than 4 h, left ventricular ejection fraction less than 50%, left ventricular hypertrophy, donor cardiac arrest, alcohol/drug abuse, coronary artery disease, recipient mechanical circulatory support, and/or elevated pulmonary vascular resistance.

In nine publications, the OCS was compared with SCS (Table 2) (14, 15, 25–31). The results of three of these studies favored OCS perfusion (27, 29, 31), including two studies that used the OCS for high-risk cases (29, 31). The other six studies did not find any significant difference in the primary outcomes (14, 15, 25, 26, 28, 30). The total preservation time was reported in five studies, and it was significantly longer in the OCS groups (14, 25, 26, 29, 30).

Botta et al. compared day-0/day-1 CK-MB levels between an OCS group and an SCS group and did not find any significant difference (26). Falk et al. compared IRI between the OCS and SCS groups by measuring interleukin (IL)-6, IL-8, IL-18, angiopoietin-2, and insulin-like growth factor-binding protein-1 immediately after and 24 h after heart transplant (27). The results showed that OCS preservation significantly reduced all these proteins. Seven studies compared short- and long-term patient survival rates and found no significant difference between the groups (14, 15, 25, 28-31).

One case report reported two long-distance heart transplantations, with or without the OCS. Although both patients remained well at 6 months with normal cardiac function, the patient who received the SCS-preserved heart had a longer hospital stay (50 vs. 12 days) and a higher cost (AU$ 234,160 vs. 56,658) compared with the OCS recipient (14). In nine publications, only the OCS was studied (Table 3) (5, 10, 16, 21, 32–35, 40). In general, the OCS preserved heart function well, resulting in a satisfactory postoperative survival rate for the recipients. Two case reports presented successful transplantations after 10 and 16 h preservation time (5, 21). In one study, hearts from both standard criteria donors and marginal donors (outside standard acceptability criteria) were preserved with the OCS, and no significant differences in 1-month, 1-year, and 2-year survival rates were found. However, there was an increased requirement for extracorporeal membrane oxygenation (ECMO) support in the standard criteria donor group (33% vs. 11%) (33).

The OCS was used for DCD hearts in 11 studies (Table 4) (17–20, 22–24, 36–39). In clinical practice, DCD hearts are retrieved with either direct procurement and perfusion (DPP) (17–19, 22–24, 36, 37, 39) or thoracoabdominal normothermic regional perfusion (TA-NRP) (20, 24, 37, 39). For DPP, after confirmation of death, a cardioplegic flush is applied. Thereafter, the heart is excised and transported in a beating state using an OCS. For TA-NRP, after confirmation of death, cardiac resuscitation is achieved with the help of an external pump. After weaning from the TA-NRP, cardiac functional assessment is performed using a pulmonary artery flotation catheter and transesophageal echocardiogram. Four studies reported comparable results between the OCS-preserved DCD hearts and the SCS-preserved DBD hearts (22, 24, 37, 39). However, two hearts were discarded after OCS preservation owing to machine failure (22). One study reported a 100% 3-month survival rate in both OCS-preserved DCD hearts and OCS-preserved marginal brain donor hearts (36). One study compared post-transplant biopsies for C4d and acute rejection episodes. The results suggested a lower IRI rate and similar patterns of cellular rejection for the OCS-preserved DCD hearts compared with the regular DBD transplantation (38). The other five publications presented successful DCD heart transplantations using OCS (17–20, 23). Messer et al. also compared the DPP plus OCS with TA-NRP plus OCS for DCD hearts and found no significant difference in 30- and 90-day survival rates (24, 37).

**TABLE 4:** Studies of normothermic machine perfusion for hearts from donation after circulatory death.

Study	Number of patients	Outcomes	Publication type
Chew et al., 2017 (36)	DCD = 12, MBD = 12	All hearts retrieved with DPP, comparable survival rate between OCS-preserved DCD hearts and OCS-preserved MBD hearts	Conference abstract
Chew et al., 2019 (22)	DCD = 23, DBD = 94	All DCD hearts retrieved with DPP, comparable survival rate between OCS-preserved DCD hearts and SCS-preserved DBD hearts	Paper
Dhital et al., 2015 (23)	DCD = 3	All hearts retrieved with DPP, survival to date: 77, 91, and 176 days	Article
Garcia et al., 2016 (17)	DCD = 2	Both hearts retrieved with DPP, survival to date: 290 and 291 days	Article
Mehta et al., 2019 (18)	DCD = 7	All hearts retrieved with DPP, 90-day survival rate 86%	Article
Messer et al., 2016 (20)	DCD = 9	8 hearts retrieved with TA-NRP + OCS; all patients survived during follow-up (range, 48–297 days)	Article
Messer et al., 2017 (24)	DCD = 26, DBD = 26	DCD hearts retrieved with DPP or TA-NRP, comparable results of the OCS-preserved DCD hearts and the SCS-preserved DBD hearts	Article
Messer et al., 2019 (37)	DCD = 50, DBD = 50	DCD hearts retrieved with DPP or TA-NRP, comparable results in 30-day survival	Conference abstract
Mohite et al., 2019 (19)	DCD = 1	Heart retrieved with DPP, alive to date at 5 months	Article
Page et al., 2017 (38)	DCD = 20, DBD = not reported	Biopsies within first month after transplantation showed significantly lower positive C4d rate in OCS-preserved DCD hearts suggesting a lower IRI rate. During first year, acute cellular rejection (2R) was lower in DCD than DBD group	Conference abstract
Page et al., 2018 (39)	DCD = 31, DBD = 31	DCD hearts retrieved with DPP or TA-NRP, comparable results	Conference abstract

DBD, donation after brainstem death; DCD, donation after circulatory death; DPP, direct procurement and perfusion; IRI, ischemia reperfusion injury; MBD, marginal brain dead; TA-NRP, normothermic regional perfusion; OCS, organ care system; SCS, static cold storage.

Five clinical trials are currently recruiting patients (Table 5) (41–45). Among these trials, three have a randomized design (42, 43, 45) and four are multicenter studies (41, 42, 44, 45). All ongoing clinical trials use patient/graft survival as the primary endpoint and patient/graft survival in a different time frame and/or graft function as secondary endpoints.

**TABLE 5:** Ongoing clinical trials.

NCT number	Institution	Study phase/design	Starting date–estimated primary completion date	Estimated number of enrolled patients	Study arms	Outcome measures (time frame)
NCT03687723 (41)	Hannover Medical School, Hannover, Germany	Multicenter, observational	October 2016–December 2021	60	Clinical use of OCS	Primary outcome: patient survival (12 months); secondary outcomes: patient and graft survival (30 days)
NCT03991923 (42)	UZ Leuven, Leuven, Flemish Brabant, Belgium, etc., total eight centers in Europe	Multicenter, randomized	July 2020–July 2021	202	NIHP, STS	Primary outcome: mortality and graft dysfunction (30 days); secondary outcomes: mortality and graft dysfunction (time frame 12 months)
NCT04066127 (43)	Skane University Hospital Lund, Skane, Sweden	Randomized	June 2020–December 2022	66	NIHP, STS	Primary outcome: survival free of acute cellular rejection and re-transplantation (12 months); secondary outcomes: I/R-tissue injury, early allograft dysfunction, and health status
NCT03835754 (44)	Cedars-Sinai, Stanford University, Yale New Haven Hospital, etc., total 12 centers from United States	Multicenter	June 2019–November 2020	48	Clinical use of OCS, high risk donors	Primary outcome: patient survival (30 days), absence of severe PGD (24 h post heart transplant); secondary outcome: patient and graft survival (30 days), incidence of severe PGD and donor heart utilization rate (24 h post-transplant)
NCT03831048 (45)	Stanford University, Yale New Haven Hospital, Mayo Clinic, etc., total 16 centers from United States	Multicenter, randomized	December 2019–August 2021	212	DCD donors: OCS, SCS	Primary outcome: survival (6 months); secondary outcome: utilization rate (within 24 h post-transplant)

DCD, donation after circulatory death; NIHP, non-ischemic hypothermic preservation; OCS, organ care system; PGD, primary graft dysfunction; SCS, static cold storage.

## Discussion

Despite encouraging results, considerable challenges still need to be overcome before sound conclusions can be drawn regarding MP for heart preservation. Existing literature in this field is limited. Most of the studies were non-randomized and retrospective, and half of the publications were conference abstracts. The total number of transplantations using MP was low, especially for HMP. A clear advantage of MP has not been observed in randomized controlled studies. Although NMP has shown its superiority in high-risk cases in non-randomized single-centre studies, high-quality clinical trials still need to be conducted.

Several publications have concluded that the effectiveness of the OCS seems to be more prominent in high-risk cases and for DCD hearts (5, 16, 46). One explanation could be that the OCS provided a platform for the functional assessment of donor hearts. During perfusion, perfusion parameters such as lactate production could be evaluated, and visual assessment could be performed. Only hearts that meet predefined criteria proceed to transplantation. However, as the only biomarker, serum lactate levels in the perfusate might not be reliable One study reported that five DCD hearts with a perfusate lactate concentration >5 mmol/L had been transplanted with a good outcome (22). As an alternative, TA-NRP can also assess DCD heart function *in situ* (24). During TA-NRP, donor hears can be assessed in a physiologic condition. With the help of a Swan-Ganz catheter and echocardiography, functional assessment can theoretically be better done during TA-NRP than OCS. In one study, two successful DCD heart transplantations were performed after TA-NRP and SCS preservation (37). However, whether the same result can be repeated for more significant number of candidates still needs to be confirmed.

MP may reduce acute graft rejection. A porcine heart study showed that NIHP could significantly reduce donor heart immunogenicity via loss of resident leukocytes, reducing recipient T cell recruitment up to 48 h following transplantation in the absence of immunosuppression (47). No clinical study has addressed on this topic so far. However, if this is confirmed clinically, all the transplantations can benefit from MP.

Ischemia is the main reason a donor heart can only be preserved within a few hours. The principle of the MP is to avoid ischemia. Both preclinical (46) and clinical (5, 21) studies have shown that successful transplantations after more than 10 h of MP preservation can be achieved. A prolonged preservation time would theoretically benefit the transplantation teams and reduce transplantation costs.

Literature on pediatric heart transplantation has been excluded in this review. As far as we know, no MP has been used for clinical pediatric heart transplantation so far. However, due to donor shortage, pediatric transplantations more often involve distant retrieval and complex operations. A MP system for pediatric donor hearts would be extra beneficial.

The perfusion technique and perfusate are the two keys to successful preservation. In Wicomb et al.’s study of HMP (9), only one of the four recipients survived over 16 months. Because the study was performed before 1982, many factors might have played roles in the low survival rate, such as the operative technique, perioperative care, etc. Among other factors, the combination of inadequate perfusion and lack of colloid in the perfusate might also have played a specific role. In pilot studies of porcine heart preserved using HMP, we observed that the albumin concentration in the perfusate was positively related to the myocardial water content (48, 49). The feasibility and effectiveness of this method have been shown in a clinical study (6). In contrast to this albumin-rich hyperoncotic and hyperkalemic solution supplemented with erythrocytes, the OCS uses diluted whole blood. This can theoretically provide all the necessary nutrients for the heart. However, some donor blood components may have adverse effects, such as pharmacological substances, metabolites, and platelets.

MP could theoretically cause hemolysis, especially at higher pressures and extended preservation times. An animal study showed no hemolysis occurred after 24 h of porcine heart perfusion with the NIHP system (49). With a higher perfusion pressure and flow, the OCS has a higher risk of hemolysis. However, we have not seen any reports about this in clinical trials. Apart from hemolysis, prolonged MP time, especially with NMP, would also lead to metabolite accumulation in the perfusate. However, with post-transplant ECMO support, successful transplantations have been reported after 10 and 16 h of total preservation time with the OCS (5, 21).

In addition to better clinical outcomes, safety and simplicity are crucially important for MP. HMP is theoretically safer and simpler to use than NMP. If a machine malfunction or user error occurs, NMP, which perfuses a beating heart, would have a narrower margin of safety. It was reported that two hearts were discarded after using the OCS owing to machine failure in one DCD study (22). In PROCEED II, five donor hearts were discarded after OCS preservation, despite these hearts being appropriate for transplantation at harvest. However, whether the OCS caused this effect was unclear (7, 50).

Using MP leads to a longer preservation time (129 min longer in the OCS group and 29 min longer in the NIHP group than in the SCS group) (6, 7). Moreover, MP requires additional surgical and technical support, proprietary equipment, appropriate transport, and additional costs. However, it may reduce the length of stay in the intensive care unit or hospital, postoperative mechanical support, and need for reoperation. Therefore, the total cost and labor demand may be reduced (14).

A challenge emerged during literature collection because the same data on MP transplantation has been used repeatedly in different conference abstracts and papers. Such examples can be found in publications from the groups of Rojas S., et al, Nilsson J., et al, Yeter R., et al, Chew, H., et al and García Sáez, D., et al. When the same data have been used in a series of publications, we included only the latest the publications and when only part of the data has been used with different study design, we included all these publications to avoid missing data (16, 33). Consequently, this may jeopardize the objectiveness of this review. Fortunately, the conclusions of these publications have been consistent, and the impact is theoretically minimal.

In summary, the machine perfusion in the form of either HMP or NMP, has emerged a potentially beneficial method for heart preservation. Based on the currently available data, when preserving a regular human donor heart, MP seems to yield clinical outcomes comparable to traditional SCS. However, HMP seems especially beneficial for high-risk cases and DCD hearts. Compared to NMP, HMP seems to be less complex, which may make it more feasible and safer, and this is an excellent advantage for the transportation of donor hearts. In future studies, we believe it’s important address the efficiency of MP for donor hearts with isolated risk factors, such as prolonged preservation time, hearts from higher age donors, or low ejection fraction. Additionally, it is also essential to develop an ideal perfusion medium for different types of MP and a system for pediatric transplantation considering the more significant donor shortage.
